# Alterations in the Gut Microbiome Composition of People Living with HIV in the Asia–Pacific Region: A Systematic Review

**DOI:** 10.3390/clinpract14030066

**Published:** 2024-05-15

**Authors:** Paul Benedic U. Salvador, Patrick Josemaria d. R. Altavas, Mark Angelo S. del Rosario, Eric David B. Ornos, Leslie Michelle M. Dalmacio

**Affiliations:** 1 Department of Biochemistry and Molecular Biology, College of Medicine, University of the Philippines Manila, Manila 1000, Philippines; pdaltavas@up.edu.ph (P.J.d.R.A.); lmdalmacio@up.edu.ph (L.M.M.D.); 2 Multi-Omics Research Program for Health, College of Medicine, University of the Philippines Manila, Manila 1000, Philippines; msdelrosario5@up.edu.ph (M.A.S.d.R.); ebornos@up.edu.ph (E.D.B.O.); 3 Department of Medical Microbiology, College of Public Health, University of the Philippines Manila, Manila 1000, Philippines

**Keywords:** human immunodeficiency virus, acquired immunodeficiency syndrome, gut microbiome, gut dysbiosis, metagenomics

## Abstract

Human immunodeficiency virus (HIV) infection continues to present a global health issue. Recent studies have explored the potential role of the gut microbiome in HIV infection for novel therapeutic approaches. We investigated the gut microbiome composition of people living with HIV (PLHIV) in the Asia–Pacific region. This review was conducted following the Preferred Reporting Items for Systematic Reviews and Meta-Analysis (PRISMA) guidelines. An electronic search was conducted in the PubMed/MEDLINE, Scopus, and ScienceDirect databases using keywords such as “HIV”, “PLHIV”, “AIDS”, “gut microbiome”, “gut dysbiosis”, and “metagenomics”. Only peer-reviewed and full-text studies published in English were included. A total of 15 studies from the Asia–Pacific region were included for analysis. Compared to healthy controls, PLHIV showed an increased abundance of Proteobacteria and its genera, which may be considered pathobionts, and decreased abundances of Bacteroidetes and several genera under Firmicutes with known short-chain fatty acid and immunoregulatory activities. Predominant taxa such as *Ruminococcaceae* and *Prevotellaceae* were also associated with clinical factors such as CD4 count, the CD4/CD8 ratio, and inflammatory cytokines. This review highlights gut microbiome changes among PLHIV in the Asia–Pacific region, indicating potential bacterial signatures for prognostication. The partial restoration of the microbiome toward beneficial taxa may ensure the long-term success of treatment, promoting immune recovery while maintaining viral load suppression.

## 1. Introduction

Human immunodeficiency virus (HIV) infection and acquired immunodeficiency syndrome (AIDS) remain major health burdens in various countries around the globe. The World Health Organization (WHO) estimated that in 2021, there were 38 million people living with HIV (PLHIV) globally, with around 1.5 million new cases of infection each year [1]. Despite advances in treatment and public health efforts, prevalence and incidence rates are rising in several regions, including North America, Africa, and Asia, leading to significant disability-adjusted life years due to comorbidities and complications associated with disease progression [2,3,4]. In Asia, the highest numbers of cases were observed in India, China, Indonesia, Thailand, and Vietnam, representing around 87% of PLHIV in the region [4]. Some countries, such as Indonesia and the Philippines, have recorded cases of epidemics, further increasing the number of new cases [4]. The persistence of HIV/AIDS globally necessitates action to enhance prevention efforts and alleviate the burden of potential complications that may arise during long-term treatment.

Novel treatment approaches are being developed, including investigations into the role of the gut microbiome in HIV infection. The gut microbiota, or the intestinal microbial community, has been shown to influence multiple physiological processes, including immune activation and homeostasis, through its interaction with intestinal immune cells [5,6]. Unfortunately, various factors such as infection or antimicrobial use may disrupt the gut microbiome, predisposing individuals to gut dysbiosis [6,7]. Previous studies have demonstrated that gut dysbiosis also occurs among PLHIV since HIV causes significant alterations in the gut microbiome such as decreased diversity, lowered abundances of bacteria producing short-chain fatty acids (SCFAs), and increased abundances of pathogenic or opportunistic bacteria [8,9,10]. Furthermore, these changes in the gut microbiome have been associated with potentially worse prognoses and treatment outcomes due to the chronic activation of systemic inflammation, poor CD4^+^ T-cell recovery, and the persistence of viral reservoirs in the immune cells of the gut [11,12,13]. In an attempt to target the gut microbiome, some studies have explored the use of probiotics such as *L. rhamnosus*, *L plantarum*, *P. acidilactici*, and *Bifidobacteria* in addition to antiretroviral therapy (ART) for PLHIV [14,15,16,17]. Probiotic supplementation has demonstrated some improvement in reducing systemic inflammation among PLHIV, as evidenced by an increase in the CD4/CD8 ratio and a decrease in cytokine levels [15,16,17]. Additionally, some studies have indicated possible benefits in reducing complications such as AIDS-related diarrhea or cardiovascular diseases through long-term supplementation with probiotics [14,17]. Despite the potential utility of probiotics and gut microbiome changes in the treatment of HIV and AIDS, there is limited research exploring the mechanisms and associations of gut dysbiosis and HIV infection.

Research on the gut microbiome and gut dysbiosis among PLHIV, particularly in the Asia–Pacific region, is limited. Given that the gut microbiome is heavily influenced by diet, there may be large variations in the results and analyses of gut dysbiosis in PLHIV based on the geographic locations of participants [5,18]. As a result, gut microbiome studies among PLHIV in other regions may have limited applicability to patients in the Asia–Pacific region. Another factor to consider for a closer investigation of the gut microbiome of PLHIV in our region is the presence of a different predominant HIV recombinant subtype, HIV-1 CRF01_AE, compared to other regions [4,19]. Different subtypes of HIV may be associated with variations in viral factors, such as replication capacity, which may affect disease progression and treatment response [20,21]. These dissimilarities in gut microbiome composition and HIV genotypes among PLHIV in the Asia–Pacific region compared to others necessitate a comprehensive analysis in this population. This systematic review aims to determine alterations in the gut microbiome composition of PLHIV in the Asia–Pacific region, identifying possible associations of gut dysbiosis with clinical factors and outcomes such as immune reconstitution and response to ART. Our goal was to identify significant changes in the predominant taxa in PLHIV in the Asia–Pacific region to gain more insight into the influence of gut dysbiosis on HIV or AIDS progression. Additionally, we aimed to explore whether these changes could be used as potential predictors for disease severity or treatment success.

## 2. Materials and Methods

### 2.1. Search Strategy and Eligibility Criteria

This systematic review was conducted following the Preferred Reporting Items for Systematic Reviews and Meta-Analysis (PRISMA) guidelines. A search for published articles was performed by two independent reviewers using the PubMed/MEDLINE, Scopus, and ScienceDirect databases. These databases were searched using keywords such as “HIV”, “PLHIV”, “AIDS”, “gut microbiome”, “gut dysbiosis”, and “metagenomics”. The PubMed interface was also searched using both free-text words and MeSH for “human immunodeficiency virus” and “gastrointestinal microbiome”. Additional keywords identified using the MeSH results for “human immunodeficiency virus” and “gastrointestinal microbiome” were also included when searching the Scopus and ScienceDirect databases. A comprehensive search was conducted, including all articles published from January 2000 to December 2023. Filters were applied to the search results where applicable, such as the date of publication, country of affiliation, and document type. Inclusion criteria were determined a priori, and these followed the PICOS framework: P, adult participants aged 18 years or older; I/E, confirmed cases of HIV infection; C, healthy or different degrees of severity of HIV infection; O, gut microbiome composition and analysis through metagenomics; and S, descriptive and experimental studies involving human participants. Only peer-reviewed, full-text studies published in English were included in this study. The exclusion criteria for this study included populations outside the Asia–Pacific region, articles containing incomplete data such as presentation abstracts or reports, studies without primary data such as review articles, and research articles that did not use stool samples or metagenomic sequencing for gut microbiome analysis.

### 2.2. Search Strategy and Eligibility Criteria

The database search results were initially screened by two independent researchers (P.J.A. and M.A.d.R.). The initial screening involved reviewing titles and abstracts to determine the suitability of studies based on inclusion and exclusion criteria. Full-text secondary screenings were also performed in duplicate by two independent researchers (P.J.A. and M.A.d.R.). Studies that did not meet all the criteria needed for this study, such as having been conducted within the Asia–Pacific region, were excluded from analysis. Disagreements between the independent researchers during the initial or secondary screening were mediated by a third independent researcher (P.B.S.).

### 2.3. Data Extraction and Quality Assessment

Data were extracted by two independent researchers (P.J.A. and M.A.d.R.) from the selected articles. These data included the (1) publication or study data (journal, authors, publication year, country of affiliation, study design, type of sample collected for gut microbiome analysis, and methods of gut microbiome analysis); (2) participant descriptions (age, sex, number of participants, HIV status or clinical staging, HIV viral load, CD4 count, treatment status, and comorbidities); and (3) effect sizes (analyses used to compare the gut microbiome between cases and controls or association studies between HIV infection and gut microbiome changes).

A quality assessment of the risk of bias in the included prospective cohort studies, cross-sectional studies, and case–control studies was performed using the Newcastle–Ottawa Scale (NOS). The NOS follows the “star system” and evaluates 8 items grouped into 3 categories: the selection of participants (a maximum of 4 stars), the comparability of the cohorts or groups (a maximum of 2 stars), and the ascertainment of outcomes (for cohort studies) or exposure (for case–control studies) (a maximum of 3 stars). Each study may receive a maximum of 10 stars; a higher score indicates better methodology and study quality. For cross-sectional studies, the adapted NOS scale created by Herzog et al. was used; this scale evaluates 7 items grouped into the same 3 categories: the selection of participants (a maximum of 5 stars), the comparability of groups (a maximum of 2 stars), and the ascertainment of outcomes (a maximum of 3 stars). Each study may receive a maximum of 10 stars, with a higher score also indicating better methodology or study quality. Two independent authors assessed the risk of bias of each individual study included in the analysis (P.J.A. and M.A.d.R.), resolving any discrepancies by consensus with a third independent researcher (P.B.S.).

## 3. Results

### 3.1. Overview

**Figure 1:**
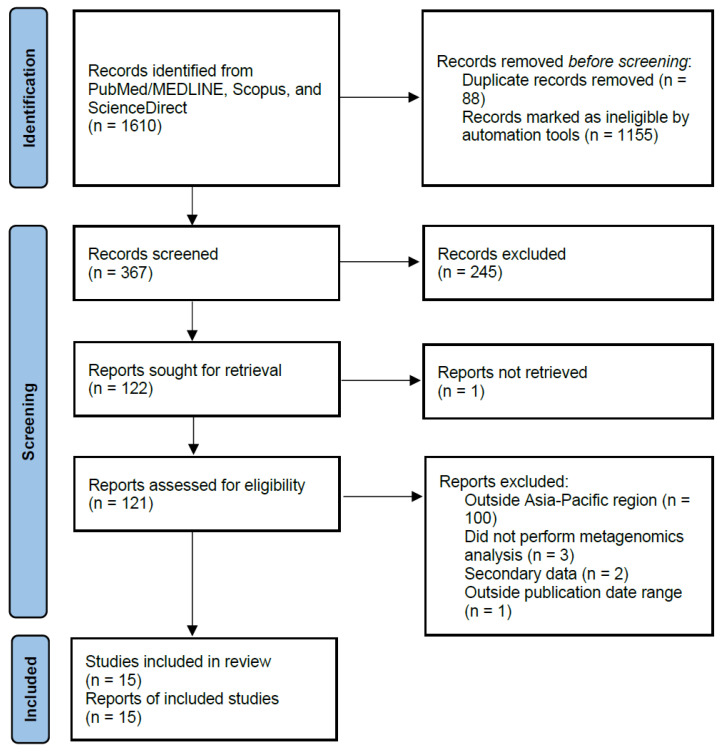
PRISMA flowchart for the identification of eligible studies.

A total of 1610 records were identified from the PubMed/MEDLINE, Scopus, and ScienceDirect databases. Filters and automations in the databases were used to remove records that did not meet the criteria for this review. Figure 1 describes the screening and selection process used in this study. A total of 1155 records were excluded due to different filters (non-English language = 10, other animals or non-humans = 123, publication type = 431, absence of keyword for HIV = 229, and affiliation outside Asia–Pacific region = 362). Of the 122 papers remaining after the first screening, most studies were excluded due to the following reasons: 100 studies had patient populations outside the Asia–Pacific region, 2 studies utilized secondary data, 3 studies did not investigate the gut microbiome composition through a metagenomics analysis of stool samples, 1 study was published in 2024, and 1 study could not be retrieved due to a lack of availability of a full-text copy.

### 3.2. Methodological Characteristics of Relevant Studies

All 15 studies selected for analysis included participants confirmed to be HIV-positive and from communities within the Asia–Pacific region. The methodological characteristics of the studies are summarized in Table 1. Geographically, most of the studies were conducted in China, accounting for 10 out of the 15 studies [22,23,24,25,26,27,28,29,30,31], while the remaining studies were divided as follows: three were from Japan [32,33,34], one was from Thailand [35], and one was from Australia [36]. Study designs comprised eight cross-sectional studies [23,24,25,26,28,31,35,36], six prospective cohort studies [22,29,30,32,33,34], and one case–control study [27]. However, two of the prospective cohort studies only employed a cross-sectional approach to describing the gut microbiomes of their participants and did not include a longitudinal analysis or a comparison of microbiome changes or associations [22,29]. All studies used stool or fecal samples for gut microbiome analyses. Similarly, all studies performed metagenomics analyses using next-generation sequencing, but there were some differences in the amplified regions: 13 studies used the 16s rRNA V3–V4 regions [23,24,25,26,27,28,29,30,32,33,34,35,36], one study used 16s rRNA V1–V3 hypervariable regions [31], and one study utilized the 16s rDNA V4 region [22]. Regarding inclusion criteria, all studies included participants confirmed to have HIV infection with a further assessment of their clinical status based on their CD4 count, HIV viral load, and/or the presence of other comorbidities. While one study did not explicitly state its inclusion criteria, the authors still recruited participants with HIV infection and recorded patient factors such as CD4 count, ART regimen, and the duration of treatment [33]. Regarding exclusion criteria, various comorbidities such as inflammatory bowel disease, hepatitis infection, malignancy, diabetes mellitus, and/or pregnancy were considered in eleven studies [22,23,24,25,26,28,30,31,32,35,36], while two studies only excluded for antibiotic or antimicrobial use [33,34], one study excluded participants with literacy and hearing or eye problems [29], and one study did not explicitly state its exclusion criteria [27]. Only nine studies explicitly stated that they excluded recent use of antibiotics, probiotics, transrectal drugs, or rectally administered enemas in time frames that ranged from 14 days to 3 months prior to participant recruitment [22,23,24,26,30,31,32,33,34]. For a comparison during analysis, healthy or non-infected controls were recruited in ten studies [22,23,24,25,27,31,32,33,34,36], five of which included age and/or sex matching for the PLHIV participants [23,31,33,34,36].

### 3.3. Risk of Bias Based on Newcastle–Ottawa Scale

All the studies selected, comprising six prospective cohort studies, eight cross-sectional studies, and one case–control study, were evaluated using the NOS. Quality assessments and ratings of all studies included in this review are summarized in the Supplementary Materials. For prospective cohort studies, Table S1 indicates that five out of six studies received a rating of 4 in the “selection” category, while one study received a rating of 3 due to a failure to report that the “outcome of interest was not present at start of study”. In the “comparability” category, all studies received a rating of 2 as they included multiple factors and confounders. In the “outcome” category, two studies received a rating of 1 since they did not address the question, “was follow-up long enough for outcomes to occur?”. Furthermore, none of the studies received a rating for the “adequacy of follow-up of cohorts” since they did not specify or quantify follow-up or the dropout rate of participants. Five studies received a total rating of 8 [30,32,33,34], one study received a total rating of 7 [29], and one study received a rating of 6 [22]. Regarding the cross-sectional studies, Table S2 summarizes the results of the quality assessments for the eight studies. In the “selection” category, seven studies received a rating of 4 due to a lack of justification for the sample size, while one study received a rating of 3 for not providing descriptions of the characteristics of non-respondents and for not justifying the sample size. In the “comparability” category, five studies received a rating of 2 and two studies received a rating of 1 for not controlling for additional factors such as comorbidities or antibiotic intake, and one study received a rating of 1 for not controlling for patient factors such as age, sex, and risk factors. For the “outcome” category, all studies received a rating of 3. Five studies received a rating of 9 [23,26,31,35,36], two studies received a rating of 8 [24,28], and one study received a rating of 7 [25]. Lastly, the quality assessment for the case–control study was conducted using the NOS and is summarized in Table S3. In the “selection” category, the study received a rating of 4. In the “comparability” category, the study received a rating of 1 for failing to control for any additional factors that may have affected the comparability of cases and controls. In the “exposure” category, the study received a rating of 3. The case–control study received a total rating of 8 [27].

### 3.4. Gut Dysbiosis and Gut Microbiome Changes among PLHIV

Significant alterations in gut microbiome composition among PLHIV were observed in the selected studies. All gut microbiota findings and associated outcomes and analyses from the studies are summarized in Table S4. Gut dysbiosis among PLHIV was observed in most studies; clustering and differentiation were observed in their bacterial communities with significant changes in the diversity and evenness of species compared to participants without HIV infection [22,23,24,25,27,28,31,32,33]. Compared to healthy controls, HIV infection has been associated with an increase in the abundances of Firmicutes [23,31] and Proteobacteria [22,23,24,31] phyla and their various genera such as *Enterococcus* [23,27,28,31], *Megamonas* [23,31,33], *Escherichia-Shigella* [24,25,28], *Streptococcus* [23,24], and *Lactobacillus* [23,27]; increases in other taxa such as *Prevotella* [25,28,31,32,33,36], Fusobacteria [24,25], and several taxa of potential pathobionts including *Moraxellaceae* [22], *Aeromonas* [27], *Pseudomonas* [27], and *Klebsiella* [25] were also observed. Compared to healthy controls, PLHIV were demonstrated to have decreased abundances of Bacteroidetes or *Bacteroides* across several studies [22,23,24,28,31,32,33] and in various genera of Firmicutes phyla such as *Faecalibacterium* [23,24,25,32], *Lachnospiraceae* and *Lachnospira* [22,23,25,32], *Ruminococcaceae* and *Ruminococcus* [22,23,25,33], *Roseburia* [22,23,24], *Alistipes* [22,24], and *Anaerostipes* [33]. Some conflicting results have been observed regarding the abundance of Actinobacteria [24,33], showing a potential an increase or decrease during HIV infection. Some studies also showed conflicting results in which the abundances of Firmicutes phyla [22,25] and *Prevotella* [23,28] decreased, highlighting the variations that may be seen in gut microbiome studies. For the effect of treatment or ART usage, four studies [23,30,31,32] investigated changes in the gut microbiota, with one study [32] comparing longitudinal changes after treatment initiation. Gut microbiome changes due to ART are conflicting as some studies have shown increases in Bacteroidetes and *Bacteroides* [23,31], Proteobacteria [30], Fusobacteria [30], *Prevotella* [31,32], and *Faecalibacterium* [23,31], while others have indicated decreases in Firmicutes [30,31], Proteobacteria [31], Bacteroidetes and *Bacteroides* [30,31,32], *Ruminococcaceae* [30], and *Faecalibacterium* [30]. An examination of the longitudinal effects of ART highlighted an increase in *Prevotella* and a decrease in *Bacteroides*, particularly with NRTI-based regimens [32]. Additionally, long-term ART has been associated with effects on the diversity of gut microbiota, such as increasing or restoring species richness and diversity, predominantly among PLHIV with low CD4 counts [23,30,33] or decreasing diversity among PLHIV with high CD4 counts [30] or those using NRTI-based regimens [32].

### 3.5. Association of Gut Microbiome Changes with Clinical Parameters among PLHIV

Multiple studies investigated changes in the gut microbiota based on clinical severity and the presence of comorbidities such as pneumocystis pneumonia (PCP) [28], neurocognitive impairment (NCI) [29], hepatitis A virus (HAV) infection [34], and hyperglycemia [35]. The results of gut microbiome associations obtained from the selected studies are summarized in Table 2. The degrees of clinical severity or the parameters of PLHIV that were included in the gut microbiome analyses were based on CD4+ and CD8+ T-cell counts [26,27,30,33], immune reconstitution [24], serum cytokine levels [25,26,31,33], the chronicity of HIV infection [36], and the route of HIV transmission [23]. Positive associations of a high CD4+ T-cell count in naïve PLHIV or good immune reconstitution during ART were seen with the abundances of *Ruminococcaceae* [24,26,27], *Bacteroidaceae* [26], *Veillonellales* [25], *Lachnospira* [27], *Escherichia-Shigella* [24], and *Alistipes* [24], while negative associations were noted for *Fusobacteriaceae* and *Fusobacterium* [24,26], *Prevotellaceae* and *Alloprevotella* [24,26], *Eubacterium* [24], *Enterobacteriaceae* and *Enterococcus* [26,27], *Prevotella_9* [28], and *Clostridiales* [25]. Conflicting results in association with CD4+ T-cell count were noted with *Faecalibacterium* [27,28] and *Roseburia* [24,27]. Pro-inflammatory responses based on T-cell count such as a high CD8+ T-cell count or a low CD4/CD8 ratio were positively associated with Proteobacteria [22], *Escherichia-Shigella* [24], *Roseburia* [24], *Veillonellaceae* [26], *Faecalibaceterium* [28], *Parabacteroides* [28], and *Prevotella_9* [28] and negatively associated with *Ruminococcaceae* [24], *Succinovibrionaceae* [26], *Faecalibacterium* [24], and *Alistipes* [24]. Moreover, serum cytokines also exhibited the following associations with gut microbiota: TNF-α was positively correlated with *Fusobacterium* and *Gammaproteobacteria* [25] and negatively correlated with *Ruminococcaceae* [25,26], and *Bacteroidales* [25], IL-2, and IL-8 are positively correlated with *Agathobacter* and negatively correlated with *Prevotellaceae* [25], IL-6 is positively correlated with *Megamonas* [31], IL-22 is positively correlated with *Dialister* [31], IFN-γ is positively correlated with *Negativiticutes* [33] and *Veillonellaceae* [33] and negatively correlated with *Clostridium XIVb* [31], and IL-19 and IL-35 are negatively correlated with *Erysipelotrichaceae* and *Atopobiaceae* [33]. Further analyses of the gut microbiome among PLHIV included associations with the functional profile and metabolites, as seen in five studies [22,27,29,31,33] and comparisons with salivary or lung microbiomes in two separate studies [28,32].

## 4. Discussion

In this systematic review, we identified the predominant taxa associated with changes in gut microbiome composition among PLHIV in the Asia–Pacific region. HIV infection is known to cause gut dysbiosis, as evidenced by significant changes not only in the diversity and evenness of the gut microbiota but also in the abundance and predominance of certain taxa [8,10]. Given the multiple factors that may affect the gut microbiota, including diet and the geographic location of participants, this review was able to determine differential abundances that may occur among PLHIV in the Asia–Pacific region based on their clinical severity, treatment status, and the presence of comorbidities.

### 4.1. Gut Dysbiosis in HIV Infection Reflects Increased Pathobionts and Decreased SCFA Producers

Our review demonstrated several key predominant taxa that may arise during HIV infection. Proteobacteria and Bacteroidetes are the primary phyla affected during HIV infection, with the former generally observed to increase while the latter decreases [22,23,24,28,31,32,33]. The phyla Firmicutes also changed significantly during HIV infection, but conflicting results were seen in four different studies [22,23,25,31]. Both studies that found an increase in the abundance of Firmicutes among PLHIV compared to healthy controls were noted to use age- and sex-matched controls, with a mean age of all participants of around 35 to 43 years old [23,31]. Previous studies showed that the abundance of Firmicutes increases with age and may be more apparent in females [37,38]. These changes may have contributed to and affected the expected changes observed during HIV infection. Aside from these phyla, the abundances of several taxa under Proteobacteria or Pseudomonadota, such as *Succinovibrionaceae*, *Moraxellaceae*, *Succinovibrio*, *Escherichia-Shigella*, *Enterobacter*, *Klebsiella*, *Pseudomonas*, and *Aeromonas*, also increased among PLHIV [22,25,27,28,31]. The observed increases in the abundances of these potential pathogens during HIV infection may signal a greater disease burden and exemplify the dysbiosis that occurs in the gut microbiome. These findings are consistent with previous studies that found higher abundances of pathobionts during HIV infection [39,40]. In addition to these, PLHIV also showed significant decreases in the abundances of Bacteroidetes, *Bacteroides*, *Bifidobacterium*, and various genera under the Firmicutes phyla including *Ruminococcaceae*, *Ruminococcus*, *Lachnospiraceae*, *Lachnospira*, *Faecalibacterium*, *Eubacterium*, *Roseburia*, *Alistipes*, and *Blautia* [22,23,24,25,28,31,32,33]. SCFA-producing genera such as *Bacteroides*, *Clostridium XIVa*, *Bifidobacterium*, *Ruminococcus*, *Lachnospira*, and *Roseburia* have been established as key mediators of gut health and development as they maintain gut homeostasis [41,42]. SCFAs ensure patent tight junctions of the gut epithelial barrier while also regulating gut hormones for nutrient absorption and local and/or systemic immunomodulation [41,43]. Reductions in various SCFA-producing bacteria in HIV infection are consistent with other studies [44,45,46]. Given the importance of SCFA producers in promoting overall health not only among PLHIV but also the general population, significant reductions in the abundances of these bacteria may be an avenue for alternative or supplementary treatment approaches.

### 4.2. Predominant Taxa as Potential Predictors of Severity and Outcomes during HIV Infection

Alterations in the composition of the gut microbiota in PLHIV may be exacerbated by their treatment status, clinical severity, and comorbidities. In this review, some predominant taxa were identified to be associated with favorable treatment outcomes or good clinical status, serving as potential predictors for prognosis among PLHIV. For instance, a high CD4 count and low CD8+ T-cell levels were both associated with *Ruminococcaceae* [24,26,27]. This immune response has been shown to have favorable outcomes due to immune regulation during the treatment of chronic HIV infection [47,48]. PLHIV on ART with a low CD4 count, high CD8+ T-cell levels, or a low CD4/CD8 ratio have been associated with poor prognosis, including residual viremia despite treatment and increased AIDS mortality [47]. Furthermore, a persistently low CD4 count and low CD4/CD8 ratio despite adequate viral suppression during ART indicate PLHIV who are considered immune or immunological non-responders (INRs) [49,50]. Compared to immune responders (IRs), INRs have a relatively worse prognosis, with an increase in the relative risk of serious non-AIDS events of around 60% and a 20% lower 15-year survival rate [49]. In this review, one study showed a significant increase in *Ruminococcus* among IRs compared to INRs and a positive association of *Ruminococcaceae* with a higher CD4 count and CD4/CD8 ratio and fewer CD8+ T-cells [24]. This is further supported by two different studies included in this review, which observed a positive association of *Ruminococcaceae* with a higher CD4 count (>200 cells/μL) among INRs and in naïve pre-AIDS PLHIV [26,27]. Another study also showed decreased levels of TNF-α, a pro-inflammatory cytokine, among PLHIV with an increased abundance of *Ruminococcaccea* [25]. These associations of *Ruminococcaceae* in PLHIV may be explained by its anti-inflammatory and immunomodulatory properties, which promote an adequate acute virological response or immune reconstitution during ART, decreasing sustained inflammation and preventing progression to AIDS [12,51]. The immunoregulatory potential of *Ruminococcaceae* and its related genus has also been demonstrated not only in HIV infection but also in other diseases such as inflammatory bowel disease, bronchial asthma, and allergy [52,53]. In terms of a worse prognosis or increased severity, this review identified *Prevotellaceae* and its genera *Prevotella_9* and *Alloprevotella* to be associated with a low CD4 count and a low CD4/CD8 ratio in three studies [24,26,28]. These results are supported by the literature, in which an increased abundance of *Prevotellaceae* and its genus *Prevotella* are observed among PLHIV and may be related to increases in inflammatory cytokines and an even earlier progression to AIDS [9,45,54,55,56]. However, multiple factors may significantly influence the abundances of *Prevotellaceae* and *Prevotella*, complicating their potential use as markers for prognosis. For instance, previous studies demonstrated *Prevotella*-rich microbiomes based on sexual practices rather than HIV infection itself [45,54,57,58]. This review also identified a positive association of *Prevotella* abundance with the chronicity of HIV infection and with the use of ART, especially with NRTI-based regimens [31,32,36]. These findings suggest that the increased abundances of *Prevotella* and *Prevotellaceae* may herald a worse prognosis due to a sustained pro-inflammatory response but may be over-represented based on sexual practices, the chronicity of HIV infection, and the use of ART regimens. Overall, changes in the abundances of *Ruminococcaceae* and *Prevotellaceae* may have the potential to become predictors of clinical status and prognosis among PLHIV in the Asia–Pacific region.

Aside from *Ruminococcaceae* and *Prevotellaceae*, multiple other taxa have been associated with HIV infection, clinical severity, and treatment status. At the phylum level, increased Firmicutes and Proteobacteria and decreased Bacteroidetes were commonly observed among PLHIV in the selected studies [22,23,24,28,31,32,33]. Studies in the literature have investigated the differential abundances of these phyla, with some considering utilizing the ratio among these taxa as prognostic markers for HIV infection and clinical severity [31,40,59,60,61]. However, conflicting results have been observed in changes in these taxa, and no clear trends have been identified from the literature or the studies included in this review [22,23,25,31,60,62]. Significant variations in changes in the abundances of these phyla may be attributed to multiple factors that may affect the predominance of these bacteria. For instance, some communities in Africa have been determined to have higher Bacteroidetes and lower Firmicutes abundances due to diet compared to communities in Europe [63]. The presence of comorbidities, particularly obesity and metabolic diseases, and the use of ART may also significantly alter the differential abundances of these phyla [44,59,60]. Nevertheless, other taxa in the altered gut microbiome composition of PLHIV may be closely followed to identify more predominant bacterial signatures associated with good outcomes and prognosis. Positive associations with a high CD4 count, high CD4/CD8 ratio, and low levels of pro-inflammatory cytokines have been identified with *Bacteroidaceae*, *Bacteroides*, *Alistipes*, and *Lachnospira*, while negative associations are seen with *Fusobacterium*, *Gammaproteobacteria*, and Proteobacteria [24,25,26,27]. Instead of focusing on the dominant phyla in the human gut microbiota, the inclusion of families and genera may be more beneficial for identifying bacterial signatures for PLHIV in the Asia–Pacific region.

### 4.3. Treatment Can Partially Restore Gut Microbial Diversity

The management of HIV infection involves the use of ART to suppress viral replication and to prevent disease progression to AIDS. However, these medications are known to cause changes in the gut microbiome of PLHIV [54,64,65,66]. This review found conflicting results regarding differences in microbial diversity between naïve PLHIV and treated participants. Some studies showed no significant differences in diversity and microbial composition, while others saw an increase or decrease in diversity and the evenness of bacterial taxa [23,24,30,31,32,33]. However, a trend emerges when changes due to ART are compared with considerations of disease severity and/or longitudinally. PLHIV on ART with a mean CD4 count of 171 cells/μL showed a significant increase in α-diversity compared to naïve PLHIV participants with a mean CD4 count of 54 cells/μL [23]. However, a comparison of microbial diversity did not show significant differences when performed between naïve and treated PLHIV with a mean CD4 count > 300 cells/μL in both groups [31]. These findings may indicate that the effect of ART in restoring or improving gut microbial diversity is more significant when dysbiosis is also greater, such as in worse cases of HIV infection. This trend of the partial restoration of diversity was also observed longitudinally in two studies when PLHIV with a low baseline CD4 count (mean and median CD4 counts of 132 and 227 cells/μL, respectively) were treated with ART [30,33]. Furthermore, the initiation of ART among PLHIV with a high baseline CD4 count (medial CD4 count = 414 cells/μL) showed a non-significant decrease in diversity [30]. Partial restoration of the gut microbiota due to ART has also been documented in the literature, in which where the effects were found to be greater among PLHIV with lower baseline gut microbial diversity or patients with worse gut dysbiosis [54,65,66]. The early initiation of ART among PLHIV, however, may produce a differential effect in which gut microbial diversity may decrease due to the direct effects of HIV treatment in the gut environment [64,65,66]. Aside from this trend in gut microbial diversity, it should be noted that almost all studies showed consensus regarding the difference in healthy and PLHIV gut microbial communities even with effective viral suppression or good immune reconstitution during long-term ART [10,23,24,27,28,31,32,33,54,64,65,66]. Consequently, efforts to ameliorate gut dysbiosis among PLHIV should not aim to completely restore microbial diversity to its pre-morbid condition. A feasible and practical outcome would be to partially restore diversity, chiefly through the promotion of beneficial taxa for immunoregulation and gut health homeostasis.

### 4.4. Study Limitations and Potential Bias in Review Process

Multiple key findings, such as potential bacterial signatures for clinical prognosis and trends in gut diversity among PLHIV in the Asia–Pacific region, have been identified in this review. Nevertheless, several limitations should be considered. For instance, variability in the results of gut microbiome studies should always be taken into account. As mentioned previously, the gut microbiome can be greatly affected by a myriad of factors. In the case of PLHIV, additional variables such as clinical status, the presence of comorbidities, and the use of antimicrobials and ART may all play a role in shaping the gut microbiome [8,9,13,40,63,66]. These variations in gut microbiome studies may be apparent in this review, as can be seen in the conflicting results of the included studies. Another limitation of this review is the inclusion of all studies among PLHIV in the Asia–Pacific region regardless of clinical status or criteria for inclusion and exclusion. Even though the demographics of the participants were controlled, there is significant heterogeneity in the studied populations. Included studies have different definitions for immune status; some use low and high CD4 counts based on WHO criteria for AIDS [26,27], while others designate cut-off values such as CD4 > 300 cells/μL for a high CD4 count [30] or a CD4 count < 250 cells/μL for a low CD4 count, CD4 count = 250 to 500 cells/μL for a medium CD4 count, and CD4 count > 500 cells/μL for a high CD4 count [33]. Some studies also controlled for recent antibiotic intake and the presence of other comorbidities, while others did not explicitly state their inclusion and exclusion criteria. Some selected studies also primarily looked at a subpopulation of PLHIV, such as those with hyperglycemia, HAV infection, or neurocognitive impairment, which limits the comparability of their results with other studies [29,34,35]. This review was also limited to the interpretation of the gut bacterial microbiome and did not consider other microorganisms that have also been shown to change among PLHIV [67,68]. Lastly, the syntheses of results in this review may be biased due to the noted heterogeneity and unmeasured confounding variables in the included studies. Inconsistencies in the studies may greatly affect the consensus and trends observed and, consequently, limit the generalizability of the findings. Nonetheless, using the corresponding NOS for different study designs, we were able to ascertain that almost all of the included studies had a minimal risk of bias and good methodological quality. These ratings indicate that the results of the individual studies are reliable despite heterogeneity and differences when compared with other studies.

## 5. Conclusions

This review investigated the gut microbiome composition of PLHIV in the Asia–Pacific region. We were able to identify *Ruminococcaceae* and *Prevotellaceae* as potential bacterial signatures for predicting HIV severity and clinical outcomes. Gut dysbiosis among PLHIV in this region was also established, with a notable increase in the abundance of Proteobacteria and various pathobionts while observing decreases in Bacteroidetes and multiple SCFA-producing genera. Moreover, ART may partially restore gut microbial diversity but will not completely address gut dysbiosis, resulting in a bacterial community that is still different from pre-morbid conditions or healthy individuals. The use of the identified predominant taxa or key bacterial signatures as predictors for PLHIV may serve as a guide for future studies to monitor these taxa and their role in HIV progression and treatment. If future studies can confirm these findings, they may also aid in the prognostication of treatment outcomes or the introduction of supplementary and alternative treatment options, such as the use of prebiotics, probiotics, postbiotics, functional food, or fecal transplantation. Trends in gut dysbiosis during HIV infection and long-term ART provide additional information on how the gut microbiome affects the acute immune response and immune reconstitution of PLHIV. Future studies may develop approaches to target gut dysbiosis in order to decrease the prevalence of INRs, ensuring immunocompetence while inhibiting the persistence of viral reservoirs. The inclusion of gut microbiome composition and gut dysbiosis in the discussion of ART and novel therapies could further improve our understanding of HIV and, ultimately, decrease the health burden caused by HIV and AIDS.

## Figures and Tables

**Table 1:** Methodological data of gut microbiome studies among PLHIV in the Asia–Pacific region between 2000–2023.

Reference	Country	Study Design	PLHIV	ART Status (Duration)	Healthy Controls	Microbiome Sample	Analysis Method
Qing et al., 2019 [22]	China	Prospective cohort	*n* = 15	Active (3 months to 5 years)	10	Stool	Illumina MiSeq; 16S rDNA v4 region
Zhou et al., 2018 [23]	China	Cross-sectional	*n =* 33 (naïve = 19; treated = 14)	Mixed (naïve or >3 months)	35 (age- and sex-matched)	Stool	Illumina HiSeq 2500; 16S rRNA V4 region
Xie et al., 2021 [24]	China	Cross-sectional	*n =* 58 (IR = 28; INR = 30)	Active (>2 years)	36	Stool	Illumina MiSeq; 16S rRNA V3–V4 region
Mingjun et al., 2022 [25]	China	Cross-sectional	*n =* 33	Not stated	28	Stool	Illumina HiSeq 2500; 16S rDNA V3–V4 regions
Lu et al., 2021 [26]	China	Cross-sectional	*n =* 34 (CD4 < 200 = 17; CD4 > 200 = 17)	Active (4 to 11 years)	None	Stool	Illumina HiSeq 2500; 16S rRNA v4 region
Zhang et al., 2023 [27]	China	Case–control	*n =* 80 (pre-AIDS = 39; AIDS = 41)	Naïve	34	Stool	Illumina MiSeq; 16S rRNA V3–V4 regions
Zhu et al., 2022 [28]	China	Cross-sectional	*n =* 83 (PCP+ = 25; PCP- = 58)	Not stated	8	Stool	Illumina MiSeq; 16S rRNA V3–V4 regions
Dong et al., 2021 [29]	China	Prospective cohort	*n =* 102 (NCI = 67; non-NCI = 35)	Active (3 to 8 years)	None	Stool	Illumina; 16S rRNA V3–V4 regions
Ji et al., 2018 [30]	China	Prospective cohort	*n =* 36 (CD4 < 300 = 14; CD4 > 300 = 22)	Active (14 to 16 months)	None	Stool	Illumina MiSeq; 16S rRNA V3–V4 regions
Ling et al., 2016 [31]	China	Cross-sectional	*n =* 67 (naïve = 32; treated = 35)	Mixed (naïve to >1 year)	16 (age- and sex-matched)	Stool	454 Life Sciences Genome Sequencer FLX system; 16S rRNA V1-V3 hypervariable regions
Imahashi et al., 2021 [32]	Japan	Prospective cohort	*n =* 20 (NRTI+ = 6; NRTI-PI− = 9; NRTI-PI+ = 5)	Mixed (naïve to >2 years)	13	Stool	Illumina MiSeq; 16S rRNA deep sequencing V3–V4 regions
Ishizaka et al., 2021 [33]	Japan	Prospective cohort	*n =* 109 (high CD4 = 61; medium CD4 = 38; low CD4 = 10)	Mixed (naïve to 15 years)	61 (age- and sex-matched)	Stool	Illumina MiSeq; 16S rRNA V3–V4 regions
Ishizaka et al., 2021 [34]	Japan	Prospective cohort	*n =* 35 (HAV+ = 10; HAV− = 25)	Active (3 to 14 years)	22 (age-matched)	Stool	Illumina MiSeq; 16S rRNA V3–V4 regions
Jayanama et al., 2022 [35]	Thailand	Cross-sectional	*n =* 40 (normoglycemia = 20; prediabetes = 20)	Active (>6 months)	None	Stool	Illumina MiSeq; 16S rRNA V3–V4 regions
Mak et al., 2021 [36]	Australia	Cross-sectional	*n =* 12 (PHI = 5; CHI = 7)	Active (4 to 23 years)	6 (age- and sex-matched)	Stool	Illumina Miseq; 16S rRNA V3 region

AIDS = acquired immunodeficiency syndrome, ART = antiretroviral therapy, CD4 = CD4+ T-cell count, CHI = chronic HIV infection; HAV = hepatitis A virus; INR = immune non-responder; IR = immune responder; NCI = neurocognitive impairment; NRTI = nucleoside reverse transcriptase inhibitor; PCP = pneumocystis pneumonia; PHI = primary HIV infection; PI = protease inhibitor; PLHIV = people living with HIV.

**Table 2:** Summary of gut microbiome changes associated with clinical parameters among PLHIV.

Clinical Parameters	Predominant Taxa or Gut Microbiota		
	Positive	Negative	Conflicting
CD4+ T-cell count	*Ruminococcaceae* [24,26,27] *Bacteroidaceae* [26] *Veillonellales* [25] *Lachnospira* [27] *Escherichia-Shigella* [24] *Alistipes* [24]	*Fusobacteriaceae* and *Fusobacterium* [24,26] *Prevotellaceae* and *Alloprevotella* [24,26] *Enterobacteriaceae* and *Enterococcus* [26,27] *Eubacterium* [24] *Prevotella_9* [28] *Clostridiales* [25]	*Faecalibacterium* [27,28] *Roseburia* [24,27]
CD8+ T-cell count	Proteobacteria [22] *Oxalobacter* [22] *Escherichia-Shigella* [24] *Roseburia* [24] *Catenibacterium* [28] *Parabacteroides* [28] *Prevotella_9* [28]	*Ruminococcaceae* [24] *Alistipes* [24]	
CD4/CD8 ratio	*Ruminococcaceae* [24] *Succinovibrionaceae* [26]	*Escherichia-Shigella* [24] *Veillonellaceae* [26] *Parabacteroides* [28] *Prevotella_9* [28] *Dialister* [28]	*Faecalibacterium* [24,28]
Plasma or serum cytokines TNF-α IL-2 and IL-8 IL-6 IL-22 IL-19 and IL-35 IFN-γ	*Fusobacterium* [25] *Gammaproteobacteria* [25] *Phascolarctobacterium* [31] *Agathobacter* [25] *Megamonas* [31] *Dialister* [31] *Negativiticutes* [33] *Veillonellaceae* [33]	*Ruminococcaceae* [25,26] *Bacteroidales* [25] *Prevotellaceae* [25] *Erysipelotrichaceae* [33] *Atopobiaceae* [33] *Clostridium XIVb* [31]	

## Data Availability

All the data included and analyzed in this study were extracted from articles that were included based on the inclusion and exclusion criteria of our study. Complete and supporting data are available from the full texts of the original articles and research.

## Supplementary Materials

The following supporting information can be downloaded at https://www.mdpi.com/article/10.3390/clinpract14030066/s1, Table S1. Quality assessment of the included prospective cohort studies using the Newcastle–Ottawa Scale. Table S2. Quality assessment of the included cross-sectional studies using the Newcastle–Ottawa Scale. Table S3. Quality assessment of the included case–control study using the Newcastle–Ottawa Scale. Table S4. Gut microbiome composition and analysis among PLHIV in the Asia–Pacific region.
