# Supplementary material for: Alterations in the Gut Microbiome Composition of People Living with HIV in the Asia–Pacific Region: A Systematic Review
Source: Clin Pract. 2024 May 15;14(3):846–61. doi: 10.3390/clinpract14030066 (PMC11130874; doi:10.3390/clinpract14030066)
Supplement: Supplementary file 1 [file clinpract-14-00066-s001.zip › clinpract-2971941-supplementary.pdf]

Supplementary Materials

Table S1. Quality assessment of the included prospective cohort studies using the Newcastle-Ottawa scale.

	Question	1	2	3	4	5	6
Selection (4)							
1	Representativeness of the Exposed Cohort						
	a) Truly representative of the average patient in the target population in the community*	*		*	*		
	b) Somewhat representative of the average patient in the target population in the community*		*			*	*
	c) Selected group of users e.g. nurses, volunteers						
	d) No description of the derivation of the cohort						
2	Selection of the Non-Exposed Cohort						
	a) Drawn from the same community as the exposed cohort*	*	*	*	*	*	*
	b) Drawn from a different source						
	c) No description of the derivation of the non-exposed cohort						
3	Ascertainment of Exposure						
	a) Secure record (eg, medical records)*	*	*	*	*	*	*
	b) Structured interview*						
	c) Written self-report						
	d) No description						
4	Demonstration that Outcome of Interest Was Not Present at Start of Study						
	a) Yes*		*	*	*	*	*
	b) No	-					
Comparability (2)							
5	Comparability of Cohorts on the Basis of the Design or Analysis						
	a) Study controls for SES (or some reasonable proxy of SES), age, race, gender*	*	*	*	*	*	*
	b) Study controls for any additional factor/s* (comorbidities, antibiotic intake or use, etc.)	*	*	*	*	*	*
	c) Inadequate degree of control						
Outcome (3)							
6	Assessment of outcome						
	a) Independent or blind assessment stated in the paper, or confirmation of the outcome by reference to secure records (x-rays, medical records, etc)*	*	*	*	*	*	*
	b) Record linkage (eg, identified through ICD codes on database records)*						
	c) Self-report (ie, no reference to original medical records or x-rays to confirm the outcome)						
	d) No description						
7	Was Follow-up Long Enough for Outcomes to Occur?						
	a) Yes (select an adequate follow up period for outcome of interest)*		*		*	*	*
	b) No	-		-			
8	Adequacy of Follow-up of Cohorts						
	a) Complete follow-up—all subjects accounted for*						

	b) Subjects lost to follow-up unlikely to introduce bias—small number lost (LESS than 10% loss-to-follow up, or description provided of those lost)*						
	c) Follow-up rate MORE than 80% and no description of those lost						
	d) No statement	-	-	-	-	-	-
Score		6	8	7	8	8	8
Quality Assessment		M	H	H	H	H	H

1. Qing et al., 2019; 2. Imahashi et al., 2021; 3. Dong et al., 2021; 4. Ishizaka et al., 2021; 5. Ji et al., 2018; 6. Ishizaka et al., 2021

* = Quality met, H = High quality, M = Moderate quality

Table S2. Quality assessment of the included cross-sectional studies using the Newcastle-Ottawa scale.

	Question	1	2	3	4	5	6	7	8
Selection (5)									
1	Representativeness of the sample								
	a) Truly representative of the average in the target population.* (all subjects or random sampling)				*		*	*	*
	b) Somewhat representative of the average in the target population.* (non-random sampling)	*	*	*		*			
	c) Selected group of users or convenience sample e.g., volunteers, members, nurses								
	d) No description of the sampling strategy								
2	Sample size								
	a) Justified and satisfactory.*								
	b) Not justified.	-	-	-	-	-	-	-	-
3	Non-respondents								
	a) Comparability between respondents and non-respondents characteristics is established, and the response rate is satisfactory.*	*	*		*	*	*	*	*
	b) The response rate is unsatisfactory, or the comparability between respondents and non-respondents is unsatisfactory.								
	c) No description of the response rate or the characteristics of the responders and the non-responders.			-					
4	Ascertainment of the exposure (risk factor)								
	a) Validated measurement tool.**	**	**	**	**	**	**	**	**
	b) Non-validated measurement tool, but the tool is available or described.*								
	c) No description of the measurement tool.								
Comparability (2)									
5	The subjects in different outcome groups are comparable, based on the study design or analysis. Confounding factors are controlled.								
	a) The study controls for the most important factor (age, sex, risk factors).*	*	*	-	*	*	*	*	*
	b) The study control for any additional factor (comorbidities, antibiotic intake).*	*	-	*	*	*	-	*	*
Outcome (3)									
6	Assessment of the outcome								
	a) Independent or blind assessment.**	**	**	**	**	**	**	**	**
	b) Record linkage.**								
	c) Self report.*								
	d) No description								
7	Statistical test								

	a) The statistical test used to analyze the data is clearly described and appropriate, and the measurement of the association is presented, including confidence intervals and the probability level (p-value).*	*	*	*	*	*	*	*	*
	b) The statistical test is not appropriate, not described or incomplete.								
Score		9	8	7	9	9	8	9	9
Quality Assessment		H	H	M	H	H	H	H	H

1. Zhou et al., 2018; 2. Xie et al., 2021; 3. Mingjun et al., 2022; 4. Lu et al., 2021; 5. Mak et al., 2021; 6. Zhu et al., 2022; 7. Ling et al., 2016; 8. Jayanama et al., 2022

* = Quality met, H = High quality, G = Moderate quality

Table S3. Quality assessment of the included case-control study using the Newcastle-Ottawa scale.

	Question	Zhang et al., 2023
Selection (4)		
1	Is the case definition adequate?	
	a) yes, with independent validation*	*
	b) yes, e.g., record linkage or based on self-reports	
	c) no description	
2	Representativeness of the cases	
	a) consecutive or obviously representative series of cases*	*
	b) potential for selection biases or not stated	
3	Selection of Controls	
	a) community controls*	*
	b) hospital controls	
	c) no description	
4	Definition of Controls	
	a) no history of diseases (endpoint)*	*
	b) no description of sources	
Comparability (2)		
5	Comparability of cases and controls on the basis of the design or analysis	
	a) study controls for SES (age, sex, etc.) *	*
	b) study controls for any additional factor (comorbidities, antibiotic use, etc.) *	-
Exposure (3)		
6	Ascertainment of exposure	
	a) secure record (e.g., surgical records)*	*
	b) structured interview where blind to case-control status*	
	c) interview not blinded to case-control status	
	d) written self-report or medical record only	
	e) no description	
7	Same method of ascertainment for cases and controls	
	a) yes*	*
	b) no	
8	Non-Response rate	
	a) same rate for both groups*	*
	b) non respondents described	
	c) rate different and no designation	
Score		8
Quality Assessment		H

* = Quality met, H = High quality

Table S4. Gut microbiome composition and analysis among PLHIV in the Asia-Pacific region.

Reference	Gut microbiota findings	Diversity analysis	Related microbiome outcomes and analyses
Qing et al., 2019[22]	PLHIV compared to healthy controls showed the following changes:<br>- Increased: Proteobacteria, *Moraxellaceae*, and *Psychrobacter* spp.<br>- Decreased: Bacteroidetes (*Rikenellaceae* and *Alistipes* spp.), Firmicutes (*Roseburia* spp., *Lachnospiraceae*, *Ruminococcaceae*) and *Microbacteriaceae*	Alpha-diversity<br>- Lower diversity among PLHIV based on Chao1 and Shannon indices but not significant compared to healthy controls	CD8+ T-cells have the following associations:<br>- Positive: Proteobacteria such as *Oxalobacer*, *Undibacterium*, *Burkholderia*, *Achromobacter*, *Ramlibacter*, and *Sphingobacteria*<br>No significant correlation between CD4+ T-cells and intestinal flora.<br><br>SCFAs such as butyric and valeric acid have the following associations:<br>- Positive: Bacteroidetes (*Rikenellaceae* and *Alistipes* spp.) and Firmicutes (*Roseburia* spp., *Lachnospiraceae*, *Ruminococcaceae*)
Zhou et al., 2018[23]	PLHIV compared to healthy controls showed the following associations:<br>- Positive: Proteobacteria and Firmicutes<br>- Negative: Bacteroidetes<br><br>Changes in population in PLHIV compared to healthy controls:<br>- Increased: Proteobacteria, *Enterococcus*, *Lachnoclostridium*, *Streptococcus*, *Lactobacillus*, *Ruminococcus* and *Streptococcus vestibularis*<br>- Decreased: Bacteroidetes, *Prevotella*, *Megamonas*, *Dialister*, *Ruminiclostridium*, *Faecalibacterium*, *Ruminococcus*, *Lachnospira*, *Roseburia*, *Blautia*, etc.<br><br>PLHIV on ART compared to naïve showed the following changes:<br>- Increased: *Bacteroides*, *Blautia* and *Faecalibacterium*	Alpha-diversity<br>- PLHIV have significantly decreased diversity based on richness estimators (PD_whole tree and observed species) and diversity index (Simpson, Shannon index)<br>- PLHIV on ART have significant increase in diversity compared to naïve based on Shannon index<br><br>Beta-diversity<br>- PLHIV with or without ART have significantly different microbiota composition compared to healthy	Route of HIV transmission may have the following associations:<br>- Homosexual transmission showed increased *Bacilli*, *Lactobacillales* and *Enterococcaceae*<br>- Heterosexual transmission showed increased abundance of *Prevotella*, *Lachnoclostridium*, *Phascolarctobacterium* and *Parabacteroides* compared with homosexual participants<br>- Intravenous drug users showed increased *Enterobacter*iales, *Enterobacteriaceae*, *Lachnospiraceae*, *Streptococcaceae*, and *Lactobacillaceae*
Xie et al., 2021[24]	PLHIV IR compared to healthy showed the following changes:<br>- Increased: Proteobacteria, Fusobacteria and Saccharibacteria phyla, *Lachnoclostridium*, *Megasphaera*, *Escherichia-Shigella*, Veillonella,	Beta-diversity<br>- Significant differentiation of bacterial communities between IR and healthy and INR and healthy using PCoA	Immune activation and response have the following associations:<br>- Nadir CD4 count is positively correlated with *Ruminococcaceae* and *Alistipes*, negatively correlated with *Fusobacterium*. and negatively associated with *Roseburia* and *Blautia*

	Streptococcus, *Fusobacterium*, and *Ruminococcus* gnavus genera<br>- Decreased: Bacteroidetes, Actinobacteria, Tenericutes and Lentisphaerae phyla, *Faecalibacterium*, *Eubacterium* rectale, *Alistipes*, *Subdoligranulum*, *Bifidobacterium*, *Roseburia*, *Ruminococcaceae* and *Parasutterella* genera<br><br>PLHIV INR compared to healthy showed the following changes:<br>- Increased: Proteobacteria, Fusobacteria, Tenericutes, Saccharibacteria; *Parasutterella*, *Megasphaera*, *Fusobacterium*, and *Ruminococcus* gnavus<br>- Decreased: *Faecalibacterium*, *Eubacterium* rectale, *Alistipes*, *Bifidobacterium*, *Blautia*, *Roseburia* and *Ruminococcaceae* genera<br><br>Both IR and INR compared to healthy showed the following changes:<br>- Increased: Proteobacteria, Fusobacteria, and Saccharibacteria<br>- Decreased: Bacteroidetes, Actinobacteria and Lentisphaerae phyla, *Faecalibacterium*, *Alistipes*, *Bifidobacterium*, *Eubacterium rectale*, and *Roseburia* genera<br><br>PLHIV IR compared to INR have the following associations:<br>- Positive: *Escherichia-Shigella*, *Blautia*, and *Ruminococcus torques* group<br>- Negative: *Eubacterium*, *Ruminoclostridium* 6, *Alloprevotella*	by weighted UniFrac matrices<br>- No significant differences between microbiota communities of IR and INR	- Current CD4 count is positively correlated with *Ruminococcaceae* and *Subdoligranulum* and negatively correlated with *Fusobacterium*<br>- CD4/CD8 ratio is positively correlated with *Faecalibacterium* and *Ruminococcaceae* and negative correlated with *Escherichia-Shigella.*<br>- CD8+ CD57+ T-cells is positively correlated with *Escherichia-Shigella,* positively associated with *Roseburia* and *Blautia*, and negatively correlated with *Ruminococcaceae* and *Alistipes*
Mingjun et al., 2022[25]	PLHIV compared to healthy have the following changes and associations:	Alpha-diversity<br>- Lower diversity among PLHIV compared to healthy	CD4 count and cytokine levels showed the following associations:

	- Increased: *Prevotella*, *Prevotella*ceae, Fusobacteria, Bacteroidales, *Klebsiella*, Succinivibrionaceae, Succinivibrio<br>- Decreased: Firmicutes, *Clostridia*, *Ruminococcaceae*, *Lachnospira*les, *Lachnospiraceae*, *Faecalibacterium*, Agathobacter<br>- *Fusobacterium* and *Escherichia-Shigella* were specific and highly abundant among PLHIV while *Subdoligranulum* was specific and highly abundant in the normal population.	based on observed species index, Chao1 index, ACE index, Shannon index, and Simpson index	- High CD4 showed increase in *Veillonellales* and *Selenomonada* and decrease in *Clostridiaceae* and *Clostridiales* compared to low CD4<br>- TNF-α is positively correlated with *Fusobacterium*_mortiferum, *Fusobacterium*, and Gammaproteobacteria and negatively correlated with *Ruminococcaceae* and Bacteroidales<br>- IL-2 and IL-8 are positively correlated with *Agathobacter* and negatively correlated with *Prevotellaceae*
Lu et al., 2021[26]	PLHIV with Low CD4 compared to high CD4 have the following changes:<br>- Increased: *Enterobacteriaceae*, *Fusobacteriaceae*, *Veillonellaceae* and *Prevotella*ceae<br>- Decreased: *Ruminococcaceae*, Succinivibrionaceae, and *Bacteroidaceae*	Alpha-diversity<br>- PLHIV with high CD4, compared to low CD4, has a significant decrease in microbial diversity based on Shannon index ($P = 0.0287$) and non-significant decrease based on Simpson index ($P = 0.0987$)<br><br>Beta-diversity<br>- There were no significant differences in microbial diversity between groups based on PCoA and NMDS analysis based on unweighted UniFrac distances	Clinical variables demonstrated the following associations:<br>- CD4 count has a positive association with *Ruminococcaceae*<br>- CD4/CD8 ratio has a positive correlation with *Succinivibrionaceae* and negative correlation with *Veillonellaceae*<br>- TNF-α has a negative association with *Ruminococcaceae*<br><br>Clinical variables and cytokine levels have the following associations:<br>- CD4 count and CD4/CD8 ratio have negative correlations with TNF-α and IL-1α<br>- CD4 T cell count has a negative correlation with MCP-1
Zhang et al., 2023[27]	PLHIV compared to healthy controls showed the following changes:<br>- Increased: *Lactobacillus*, *Enterococcus*, *Brevundimonas*, *Aeromonas* and *Pseudomonas*<br><br>AIDS compared to pre-AIDS PLHIV showed the following associations:	Beta-diversity<br>- Significant differences in the microbial diversity among AIDS and pre-AIDS based on PCoA with Bray-Curtis dissimilarity and PERMANOVA	Disease severity and metabolites showed the following correlations:<br>- L-tryptophan is positively correlated with *Enterococcus*, *Enterococcus durans* and *Lactobacillus* among PLHIV<br>- Phenylethylamine is positively correlated with *Enterococcus* and *Enterococcus durans*

	- Positive: *Enterococcus* and *Lactobacillus*<br>- Negative: *Faecalibacterium*, *Lachnospira*, *Ruminococcaceae*_UCG-002, *Roseburia* and *Dorea*	- Gut microbial diversity in different stages of HIV infection can be clustered in three distinct clusters based on PLS-DA	- Niacinamide and fumaric acid are positively correlated with *Fusicatenibacter*<br>- Pyridoxine is positively correlated with *Bacteroides plebeius*
Zhu et al., 2022[28]	HIV infection compared to healthy controls showed the following changes:<br>- Increased: g_*Enterococcus*, g_*Escherichia-Shigella*, and g_*Erysipelatoclostridium*<br>- Decreased: g_Betaproteobacteria_unclassified, g_*Prevotella*, g_Bacteroidetes_unclassified, g_Mitochondria_unclassified<br><br>PCP comorbidity showed the following changes:<br>- Increased: g_*Prevotella_9*, g_*Holdemanella*, g_*Catenibacterium*, and g_*Escherichia-Shigella*	Alpha-diversity<br>- No observed differences in diversity based on Chao1, Observed species, Goods_coverage, Shannon, Simpson, and other indices.<br><br>Beta-diversity<br>- Unweighted pair group method with arithmetic mean (UPGMA) analysis showed that samples formed two large clusters, namely different intestinal types	Clinical variables have the following correlations:<br>- CD4 count is negatively correlated with g_*Faecalibacterium*, g_*Dialister*, g_*Prevotella_9*, g_*Parabacteroides*<br>- CD8 is positively correlated with g_*Catenibacterium*, g_*Parabacteroides*, g_*Prevotella_9*<br>- CD4/CD8 ratio is negatively correlated with g_*Faecalibacterium*, g_*Prevotella_9*, g_*Dialister*, g_*Parabacteroides*<br>- WBC count is positively correlated with g_*Blautia*, g_*Megamonas*, g_*Parabacteroides*, g_Firmicutes_unclassified, g_*Eggerthella* and g_*Phascolarctobacterium* and negatively correlated with g_*Erysipelatoclostridium* and g_*Sutterella*<br><br>Lung and gut microbiota composition showed the following findings:<br>- Phylum level showed that *Firmicutes* were the most abundant component in the intestinal tract of HIV-infected patients (70.19%), and the relative abundance of *Firmicutes* in the lung was also high (30.48%; the most abundant *Proteobacteria* was 35.97%)<br>- *Firmicutes* was the most abundant component in the intestinal tract (40.54%) in HIV+ (PCP-) patients, and the relative abundance of *Firmicutes* in the lung was also high (15.75%; the most abundant *Proteobacteria* was 58.42%)<br>- *Firmicutes* was the most abundant component in the intestine of PCP+ patients (38.33%), and the relative abundance of *Firmicutes* in lungs was also high (28.24%; the abundance of *Proteobacteria* was 55.48%)<br>- Genus-level sequencing analysis showed that *Streptococcus*, *Enterococcus*, and *Veillonella* were the most abundant genera in the gut and lung

Dong et al., 2021[29]	NCI compared to non-NCI PLHIV showed the following changes:<br>- Increased: Spirochaetes and Epsilonbacteraeota phyla and *Klebsiella*, *Alloprevotella*, *Catenibacterium*, Coriobacteriales, *Streptococcus*, Lactobacilalles, and Campylobacterales<br>- Decreased: *Succinovibrio*, *Faecalibacterium*, *Ruminococcus*_1, *Coprococcus*_2, and Bacteroidales_RF16_group genera	Alpha-diversity<br>- No significant difference between the NCI and non-NCI groups based on Shannon, Simpson, and Chao indices<br><br>Beta-diversity<br>- No significant differences in the microbial communities of both groups based on unweighted and weighted UniFrac distance matrices	Clinical parameters showed the following correlations:<br>- CD4 count is inversely correlated with *Streptococcus* and *Treponema_2*<br>- Left CIMT is positively correlated with *Klebsiella* and inversely correlated with *Faecalibacterium*, *Ruminococcus*_1, *Coprococcus*_2, and *Ruminococcaceae*_NK4A214_group<br>- Right CIMT is positively correlated with *Klebsiella* and inversely correlated with *Faecalibacterium*<br><br>Gut metabolites and plasma 25(OH)D have the following associations:<br>- Bile acids, glycerophosphoinositols, fatty acids, eicosanoids, and fatty amides are positively correlated with *Klebsiella* and negatively correlated with *Faecalibacterium*, *Ruminococcus_1*, and *Coprococcus_2*<br>- Plasma 25(OH)D is positively associated with *Faecalibacterium*, Corprococcus_2, and *Ruminococcaceae*_NK4A214_groups
Ji et al., 2018[30]	Use of ART among PLHIV showed the following changes:<br>- Increased: Proteobacteria and Fusobacteria phyla, Gammaproteobacteria and Fusobacteria classes, *Enterobacter*iales and Fusobacteriales orders, *Enterobacteriaceae* and *Fusobacteriaceae* families, and *Klebsiella* and *Fusobacterium* genera<br>- Decreased: Bacteroidetes (Bacteroidia class and Bacteroidales order) and Firmicutes (*Ruminococcaceae* and *Faecalibacterium*)<br><br>Use of ART among PLHIV with low CD4 count showed the following changes:<br>- Increased: Bacillales (Family_XII_o_Bacillales and *Exiguobacterium*)	Alpha-diversity<br>- PLHIV with low CD4 count compared to those with high CD4 count have significantly lower diversity based on Shannon and Simpson indices<br>- ART among PLHIV with low CD4 count significantly restored the diversity based on Shannon and Simpson indices<br>- ART among PLHIV with high CD4 count caused non-significant decrease in diversity<br><br>Beta-diversity	

		- Microbial community profiles among PLHIV before and during treatment with ART are statistically distinct based on Adonis analysis of weighted UniFrac distance metric and principal-coordinate analysis	
Ling et al., 2016[31]	PLHIV compared to healthy controls showed the following changes:<br>- Increased: Firmicutes and Proteobacteria phyla, *Prevotella*, *Faecalibacterium*, Phascolarctobacterium, Butyricicoccus, *Erysipelotrichaceae* incertae sedis, *Catenibacterium*, *Dorea*, *Enterobacter*, *Enterococcus* and *Megamonas* genera<br>- Decreased: Bacteroidetes phyla, *Bacteroides*, *Dialister*, *Clostridium* XIVa, *Clostridium* XIVb, Barnesiella and *Coprococcus* genera<br><br>Use of ART among PLHIV showed the following changes:<br>- Increased: Bacteroidetes and Synergistetes phyla, *Prevotella*, *Faecalibacterium*, *Alistipes*, Oscillibacter, Barnesiella, *Dialister* and Odoribacter genera<br>- Decreased: Firmicutes and Proteobacteria phyla, *Megamonas*, Veillonella, *Blautia*, *Clostridium* XVIII and *Enterococcus* genera	Alpha-diversity<br>- No statistically significant differences between the microbial diversity among PLHIV and healthy controls based on Shannon and Simpson indices<br><br>Beta-diversity<br>- Microbial communities among PLHIV are significantly different compared to healthy controls based on principal coordinate analysis<br>- No statistical difference in microbial communities of untreated and treated PLHIV based on unweighted UniFrac analysis	Inflammatory cytokines have the following correlations:<br>- TNF-α is positively correlated with *Phascolarctobacterium*<br>- IL-6 is positively correlated with *Megamonas*<br>- IL-22 is positively correlated with *Dialister*<br>- IFN-γ is negatively correlated with *Clostridium XIVb*
Imahashi et al., 2021[32]	PLHIV compared to healthy controls showed the following changes:<br>- Increased: *Prevotella*<br>- Decreased: *Bacteroides*, *Faecalibacterium*, and *Lachnospiraceae*<br><br>Long-term ART was associated with the following:	Alpha-diversity<br>- PLHIV has increased species-richness estimates (Chao1 indices) compared to healthy<br>- Noted decreased diversity over time in ART; inversely	Salivary microbiome of PLHIV showed the following:<br>- No significant differences in *Prevotella*, *Streptococcus*, and *Veillonella* compared to healthy<br>- No noted increase in periodontal-disease-related genera such as Porphyromonas, Actinomyces, Treponema, Aggregatibacter, Shuttleworthia, Gemella, *Dialister*, and Granulicatella<br>- No significant changes in diversity compared to healthy

	- ART especially NRTI-based regimens result in higher changes in abundance in increase of *Prevotella* and decrease in *Bacteroides*<br>- Time-course increase in *Succinivibrio* and *Megasphaera*. No increase in *Clostridium*	correlated with ART especially NRTI(+) regimens<br><br>Beta-diversity<br>- PLHIV especially those using NRTI-based regimen has increased beta-diversity based on weighted UniFrac distances compared to healthy controls	
Ishizaka et al., 2021[33]	High CD4 PLHIV compared to healthy controls showed the following changes:<br>- Increased: Actinobacteria, *Prevotella*, Negativicutes (*Dialister*, *Megamonas*, and *Acidaminococcaceae*), Coriobacteriia (*Collinsella* and *Slackia*), and Bacilli (*Catenibacterium* and *Holdemanella*)<br>- Decreased: *Bacteroides* and *Clostridia* (*Ruminococcaceae* and *Anaerostipes*)	Alpha-diversity<br>- Low CD4 PLHIV compared to healthy controls have decreased diversity based on Shannon index<br>- ART showed varying degrees of restoration of bacterial richness based on Shannon index<br><br>Beta-diversity<br>- Bacterial communities between high CD4 and healthy controls are statistically significant based on Weighted UniFrac distance and its principal-coordinate analysis	Functional microbiome profiles showed the following associations:<br>- *Eggerthella* (class *Coriobacteriia*) and *Holdemanella* (class *Bacilli*) are directly associated with elevated truglycerides and decreased high-density lipoproteins<br><br>Patient and clinical parameters have the following associations:<br>- Age is positively correlated with class *Coriobacteriia* and families *Coriobacteriaceae* and *Eggerthellaceae* among healthy controls but not among PLHIV<br>- IFN-γ, IL-4, and IL-1β are positively correlated with Negativicutes (Acidaminococcales, *Acidaminococcaceae*, and Veillonellaceae)<br>- Chemokines targeting monocytes are positively correlated with *Erysipelotrichales*, and some under *Negativicutes*<br>- IL-19 and IL-35 are negatively correlated with families *Erysipelotrichaceae* in *Bacilli* and *Atopobiaceae* in *Coriobacteriia*
Ishizaka et al., 2021[34]	HAV coinfection among PLHIV showed the following changes:<br>- Increase at onset: Fusobacteria and Desulfobacterota<br>- Changes over-time: increased Actinobacteria or Bacteroidota (*Bifidobacterium* and *Bacteroides*) and decreased Proteobacteria	Alpha-diversity<br>- Significant transient elevations followed by decrease in diversity were observed during HAV coinfection based on observed OTUs and Shannon index	

	- At phylum level, microbiome composition became similar to PLHIV without HAV infection after 100 days		
Jayanama et al., 2022[35]	Prediabetes PLHIV compared to normoglycemia PLHIV showed the following changes:<br>- Increased: Firmicutes (*Streptococcus* and Anaerostignum)<br>- Decreased: Firmicutes, Bacteroidota, Cyanobacteria, Desulfobacerota, Verrucomicrobiota, *Akkermansia*, Gastranaerophilales, *Desulfovibrio*, *Butyricimonas*, *Colidextribacter*, *Christensenellaceae* R 7 group, *Victivallis*, Uncultured Bacteroidota, Uncultured phylum Firmicutes, *Holdemanella*, UCG-005, *Eubacterium ruminantium* group, and family *Oscillospiraceae*-associated group	Alpha-diversity<br>- Significantly lower diversity among pre-diabetes PLHIV compared to normoglycemia PLHIV based on Shannon index, Faith's phylogenetic diversity, observed OTUs richness, and Evenness<br><br>Beta-diversity<br>- Microbial communities between normoglycemia and pre-diabetes PLHIV have significant clustering based on PerMANOVA of unweighted UniFrac but is not significant based on Bray-Curtis, Jaccard, and weighted UniFrac	
Mak et al., 2021[36]	Both CHI and PHI have the following changes:<br>- High abundance of *Faecalibacterium*, *Prevotella*, and *Bacteroides*<br><br>PHI compared to CHI showed the following changes:<br>- Increase: *Spirochaeta* spp.<br>- CHI has a non-significant increase in *Acidaminococcus* spp., increasing abundance of *Prevotella*, and decreasing *Faecalibacterium*	Alpha-diversity<br>- More even distribution in microbiome of healthy compared to PLHIV based on Simpson index<br>- PLHIV has a non-significant decreasing trend in species richness based on Shannon diversity index H’<br><br>Beta-diversity<br>- No significant difference between groups in overall microbiota composition	

		based on Bray Curtis dissimilarity, as assessed by PERMANOVA	

AIDS = acquired immunodeficiency syndrome, ART = antiretroviral therapy, CD4 = CD4+ T-cell count, CHI = chronic HIV infection, CIMT = carotid intima-media thickness, HAV = hepatitis A virus INR = immune non-responders, IR = immune responders, NCI = neurocognitive impairment, NRTI = nucleoside reverse transcriptase inhibitor, PCP = pneumocystis pneumonia, PHI = primary HIV infection, PI = protease inhibitor, PLHIV = people living with HIV, SCFA = short-chain fatty acids
